# FlgJ cell-wall hydrolyzing activity enhances, but is not required, for flagellum assembly in *Salmonella enterica*

**DOI:** 10.1128/jb.00238-26

**Published:** 2026-08-19

**Authors:** Yann H. U. Chevance, Miki Kinoshita, Fabienne F. V. Chevance, Tohru Minamino, Keiichi Namba, Kelly T. Hughes

**Affiliations:** 1 The School of Biological Sciences, The University of Utah 172838, Salt Lake City, Utah, USA; 2 Graduate School of Frontier Biosciences, The University of Osaka, Suita, Osaka, Japan; 3 JEOL Yokogushi, Research Alliance Laboratories, The University of Osaka, Suita, Osaka, Japan; National Institutes of Health, Bethesda, Maryland, USA

**Keywords:** flagella, cell wall penetration, *Salmonella*

## Abstract

Penetration of the peptidoglycan (PG) layer by the nascent flagellar rod is a critical step in basal body assembly and has long been attributed to the acetylglucosaminidase activity of the flagellar rod cap protein FlgJ. Previous work in *Salmonella enterica* suggested that occasional preexisting openings in the PG layer allow some flagella to assemble in the absence of the FlgJ enzymatic activity. More recently, studies in *Bacillus subtilis* demonstrated that membrane mobility of nascent flagellar structures enables rod penetration without dedicated PG hydrolysis. Here, we revisited the requirement for FlgJ acetylglucosaminidase activity in *S. enterica* by testing whether inhibition of class 3 flagellar gene expression by the anti-σ28 factor FlgM contributes to the flagellation defect of FlgJ catalytic mutants. Consistent with previous studies, loss of FlgJ acetylglucosaminidase activity did not abolish flagellar assembly but instead reduced its efficiency, resulting in a heterogeneous population in which many cells assembled functional basal bodies and flagella. Deletion of *flgM* significantly increased both the proportion of flagellated cells and the number of flagellar filaments per cell, indicating that reduced class three gene expression contributes substantially to the observed defect. These findings support a model in which FlgJ enzymatic activity enhances the efficiency of local PG remodeling but is not essential for rod penetration or basal body assembly. Our results demonstrate that acetylglucosaminidase-independent flagellar rod growth occurs in *S. enterica*, although less efficiently than in organisms that lack FlgJ-like PG hydrolases, highlighting the evolutionary diversity of mechanisms that accommodate flagellar assembly through the bacterial cell wall.

**IMPORTANCE:**

Bacterial flagella allow pathogens, such as *Salmonella*, to navigate complex environments and invade host cells. During flagellar assembly, the basal body must traverse the peptidoglycan layer, a step-long thought to require the dedicated cell wall-degrading activity of FlgJ. Here, we show that *Salmonella* can assemble functional flagella, even when the acetylglucosaminidase activity of FlgJ is genetically inactivated. Although these mutants produce fewer flagella per cell, the assembly pathway remains active, and enhanced σ^28^-dependent gene expression partially restores flagellation. These findings reveal that early steps in flagellar assembly are more diverse than previously thought and that differences in cell wall structure between gram-positive and -negative bacteria likely determine whether a cell wall hydrolyzing activity is required for flagellum assembly.

## INTRODUCTION

The bacterial flagellum is a complex nanomachine that enables motility and plays an important role in *Salmonella* pathogenesis. In gram-negative bacteria, flagellar assembly requires the coordinated construction of membrane-spanning and periplasmic structures, culminating in the formation of the basal body, hook, and filament. A key challenge during early flagellar assembly is the penetration of the peptidoglycan (PG) layer by the nascent rod. In enteric bacteria, such as *Salmonella enterica* and *Escherichia coli*, this process has been attributed to FlgJ, a bifunctional periplasmic protein composed of an N-terminal scaffolding domain required for rod assembly and a cell wall-hydrolyzing activity C-terminal domain proposed to locally hydrolyze PG allowing rod assembly through the PG layer (1, 2).

**Fig 1:**
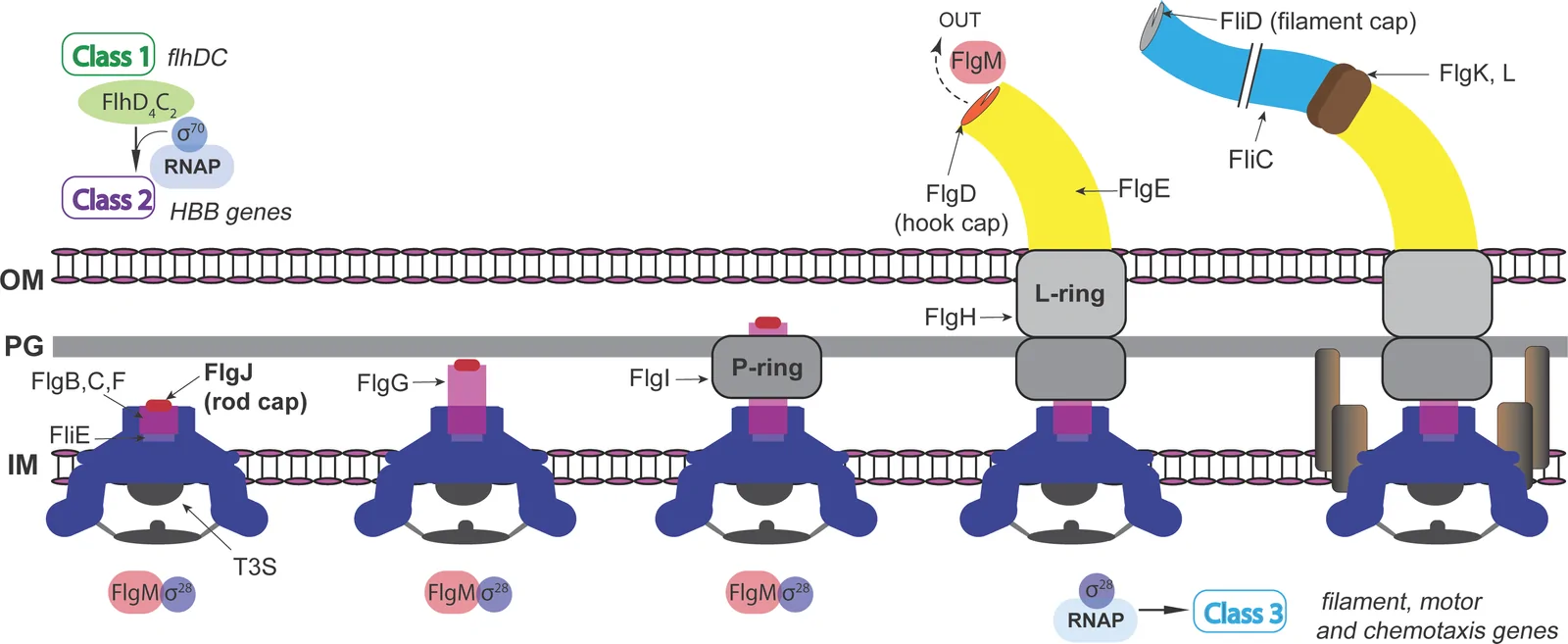
Flagellar rod assembly pathway in *Salmonella*. Rod subunits include an adaptor (FliE), a proximal rod (FlgB, FlgC, and FlgF), and a distal rod (FlgG). It is not known whether the FlgJ rod cap assembles before or after proximal rod assembly. FlgG distal rod subunits are secreted through the type III secretion apparatus (T3S) and polymerize beneath the transient FlgJ rod. If the growing rod encounters any PG barrier to further polymerization, then FlgJ is believed to help the rod continue to traverse through peptidoglycan layer (PG) due to its acetylglucosaminidase activity. Rod polymerization ceases when it reaches the outer membrane. The assembly of the PL-ring bushing at the rod tip dislodges the FlgJ rod cap and simultaneously forms a pore in the outer membrane. The FlgD hook cap then assembles at the rod tip and acts as a scaffold for hook subunit polymerization until the hook reaches its terminal length of ~55 nm. Hook length is determined by a secreted molecular ruler FliK (not shown) that catalyzes the flagellar T3S specificity switch upon hook length completion, resulting in a cessation of early, rod-hook subunit secretion and the initiation of late flagellar substrate secretion, which is mostly the subunits that make to the long external filament structure.

In *Salmonella*, flagellar genes are regulated in a transcriptional hierarchy of three promoter classes that couple gene regulation to assembly (reviewed in reference 3). At the top of the transcriptional hierarchy is the class 1 *flhDC* operon, which encodes a transcriptional activator complex needed for expression of the flagellar class 2 promoters (Fig. 1). The class 2 promoters direct the transcription of genes needed for the structure and assembly of the hook-basal body (HBB) structure and two key regulatory genes, *flgM* and *fliA*. The *fliA* gene encodes a flagellar-specific transcription factor, σ,^28^ that directs RNA polymerase to class 3 promoters for transcription of genes required for filament formation, motor rotation, and the chemosensory system. FlgM is an anti-σ^28^ factor that prevents σ^28^-dependent class 3 transcription prior to HBB completion. Prior to HBB completion, the flagellar T3S system is specific for rod-hook protein substrates. Upon HBB completion, the flagellar T3S apparatus undergoes a secretion-specificity switch where rod-hook substrates are no longer recognized, and the secretion apparatus is specific for late secretion substrates, including FlgM and proteins needed for filament assembly. Secretion of FlgM releases σ^28^ to transcribe class 3 promoters upon HBB completion.

Flagellum assembly begins with the construction of an inner membrane-embedded complex that includes a type III secretion system for the secretion of flagellar-specific substrates from the cytoplasm (Fig. 1, reviewed in references 4 and 5). Secreted proteins make up the rod-hook-filament axial structure that extends from the inner membrane. The structure is hollow, and secreted subunits travel through its center and self-assemble at the growing tip. From the inner membrane, the rod assembles through the cell wall and periplasmic space to the outer membrane. The rod acts as a drive shaft for the flagellar motor, and its growth terminates upon reaching the outer membrane. Outer membrane penetration requires the formation of the PL-ring bushing (6). The proteins required for PL-ring assembly are secreted through the general Sec secretion system. Thus, rod completion and PL-ring completion are uncoupled steps in flagellum assembly. The P-ring forms first around the distal rod. Assembly of the L-ring on the P-ring produces the outer membrane pore necessary for continued flagellum assembly from the cell surface. Completion of the PL-ring coincides with the loss of the rod cap, protein FlgJ, and its replacement with the hook cap (7). The hook extends from the cell surface ~55 nm. Hook completion is followed by assembly of hook-filament junction proteins and the replacement of the hook cap with the filament cap. Filaments now polymerize under the filament cap to a length of ~10 µm from the cell surface. Filament length is self-limited by hindered diffusion of secreted substrates through its central channel (8). The longer the filament, the more difficult it is for secreted subunits to reach the tip.

The PG-hydrolyzing activity of FlgJ was initially characterized as a muramidase but was later shown to function as an acetylglucosaminidase (2, 9). The conserved residues E223 and D248 form part of the catalytic center shared with muramidase-family enzymes, and substitution of these residues (E223Q/D248N) significantly reduced FlgJ-mediated peptidoglycan hydrolysis (2). Early studies using this double mutant reported the formation of basal bodies lacking L-rings, leading to the model that efficient PG digestion is required for the distal rod to reach the outer membrane and support L-ring assembly (1). EM analysis of basal structures in *flgJ* PG-hydrolyzing defective mutants revealed structures with rods and P-rings, suggesting that P-ring formation occurs independently of complete periplasmic traversal. Thus, if the PG-hydrolyzing activity of FlgJ was defective, the growing rod structure could not reach the outer membrane to complete PL-ring formation and outer membrane penetration.

Comparative genomics challenges this model. Many gram-positive bacteria, including *Bacillus subtilis*, lack a FlgJ-type muramidase despite possessing a substantially thicker peptidoglycan (PG) layer, yet they assemble functional flagella efficiently (10). These observations suggest that alternative hydrolases, more distributed mechanisms of PG remodeling, or placement of nascent basal structures at positions where the rod can polymerize through a porous region in the cell wall may support rod penetration in the absence of a dedicated FlgJ-like enzyme. In this context, PG remodeling refers to the localized and dynamic enzymatic modification of the cell wall, including cleavage of glycan strands, hydrolysis of peptide crosslinks, and incorporation of newly synthesized material. These processes collectively alter the structure and porosity of the PG layer, creating transient opportunities for large macromolecular complexes, such as the flagellar rod, to traverse the cell wall. Alternatively, the physical properties of a more porous gram-positive cell wall may reduce the requirement for a dedicated hydrolytic activity. Recent work proposes that in *B. subtilis*, basal bodies remain mobile within the membrane during early assembly, allowing the growing rod to probe the PG network for pores of sufficient size to permit passage (11). Upon encountering a permissive opening, the rod traverses the PG layer and assembly proceeds without further basal body movement. This model proposes a fundamentally different strategy for overcoming the PG barrier, emphasizing physical exploration of a heterogeneous cell wall rather than reliance on localized enzymatic cleavage.

The original observations that FlgJ was essential to cell wall penetration by the flagellar rod in *Salmonella* were made using high-level expression from plasmids (1, 2), raising concerns about stoichiometry, envelope stress, and altered regulation. Because periplasmic hydrolases can be dosage-sensitive, chromosomal reconstructions are necessary to define the physiological requirement for FlgJ’s peptidoglycan hydrolyzing activity. Here, we reexamine the requirement for FlgJ-mediated PG hydrolysis using chromosomally encoded catalytic mutants in *Salmonella*. An acetylglucosaminidase-deficient allele *flgJ* (E223Q, D248N) expressed from its native chromosomal locus exhibited greater motility and flagellar assembly than previously reported (1, 2), with approximately 50% of cells producing surface-exposed flagella. While one of the previous studies considered the effect of FlgM accumulation in strains defective in FlgJ PG-hydrolyzing activity on flagellum assembly, it was not directly tested. Our results show that increased σ²⁸-dependent gene expression by deletion of the anti-σ²⁸ factor FlgM did partially compensate for defects associated with the *flgJ* mutant. Fully formed basal bodies were also observed in the absence of FlgJ catalytic activity. Together, these findings demonstrate that FlgJ acetylglucosaminidase activity enhances, but is not strictly required for, rod penetration of the PG layer and subsequent outer membrane ring assembly in *Salmonella*. These results suggest that flagellar rod assembly can proceed without FlgJ-mediated hydrolysis, likely by exploiting transient or preexisting discontinuities in the PG network.

## MATERIALS AND METHODS

### Bacterial strains and growth conditions

All strains used in this study are derivatives of *Salmonella enterica* serovar Typhimurium LT2 (Table 1). Strains were grown in lysogeny broth (LB) (10 g tryptone, 5 g yeast extract, 5 g NaCl per liter) with aeration or on solid media (LB supplemented with 12 g/l Apex agar) at 37°C. When appropriate, antibiotics were added to LB media for selections or plasmid retention at the following final concentrations: 12.5 µg/mL chloramphenicol (Cm) and 15 µg/mL tetracycline-HCl (Tc). Expression of *flhDC* from the tetracycline promoter (TPOP) was induced with anhydrotetracycline (ATc; 125 ng/mL).

**TABLE 1:** Strains used in this study

Strain	Genotype
TH437	LT2 (John Roth)
TH5139	∆*flgM5628*::FRT
TH8095	*rrfB*::*tetRA (E. coli*)
TH13962	∆*flgJ7664* (∆ amino acids 6 through 311 [of 316])
TH24874	*fliC6500*(T237C) *fliM5978*::GFP2 ∆*hin-5717*::FRT
TH29885	*flgJ9452*::t*etRA*
TH29890	*flgJ9454* (E223Q D248N)
TH29920	*flgJ9454 fliC6500*(T237C) *fliM5978*::GFP2 ∆*hin-5717*::FRT
TH30112	∆*flgM5628*::FRT *fliC6500*(T237C) *fliM5978*::GFP2 ∆*hin-5717*::FRT
TH30113	∆*flgM5628*::FRT *flgJ9454 fliC6500*(T237C) *fliM5978*::GFP2 ∆*hin-5717*::FRT
TH30143	∆*flgM5628*::FRT *flgJ9454*
TH30157	*attB*::CmR-P_fliF_-YFP ∆*galK*::ApR-P_fliC_-CFP
TH30158	*flgJ9454 attB*::CmR-P_fliF_-YFP ∆*galK*::ApR-P_fliC_-CFP
TH30159	∆*flgM5628*::FRT *attB*::CmR-P_fliF_-YFP ∆*galK*::ApR-P_fliC_-CFP
TH30160	∆*flgM5628*::FRT *flgJ9454 attB*::CmR-P_fliF_-YFP ∆*galK*::ApR-P_fliC_-CFP
TH30407	*flgM5628*::FRT ∆*flgJ7664*
TH30535	*P*(*flhDC*)*5451*::Tn*10d*Tc[del-25] ∆*hin-5718*::FRT *fljB5001*::MudJ
TH30536	*P*(*flhDC*)*5451*::Tn*10d*Tc[del-25] ∆*hin-5718*::FRT *fljB5001*::MudJ *∆flgJ7664*
TH30537	*P*(*flhDC*)*5451*::Tn*10d*Tc[del-25] ∆*hin-5718*::FRT *fljB5001*::MudJ *flgJ9454*
TH30538	*P*(*flhDC*)*5451*::Tn*10d*Tc[del-25] ∆*hin-5718*::FRT *fljB5001*::MudJ *flgJ9454*∆*flgM5628*::FRT

### Construction of *flgJ* alleles

Double-amino acid substitution mutation in FlgJ (E223Q D248N) was generated using λ-Red recombineering using plasmid helper pSim5 (Cmᴿ) to insert and remove a *tetRA* element derived from transposon Tn*10* as described (12). PCR products were amplified from genomic DNA from *E.coli* strain TH8095 with primers flgJ-222-term-tetA (cgccagttggaaagggccggtgacggagatcacca ccactAGAGTAGGGAACTGCCAG) and flgJ-248-tetR (cggcagcgtagcgtgggttacgcgttaacagcgcga cataTTAAGACCCACTTTCACATT) to amplify the *flgJ*::terminator *tet*A-R cassette or from *Salmonella enterica* serovar Typhimurium LT2 strain with primers flgJ-E223Q (cgccagttggaaaggg ccggtgacggagatcaccaccactCAGtacgaaaatggcgaagcga) and flgJ-D248N (cggcagcgtagcgtgggttacgc gttaacagcgcgacataGTTcgataatgcctccagatacg) to produce the *flgJ* E223Q D248N DNA PCR fragment. Phusion polymerase was used to amplify these PCR reactions. The *flgJ*::terminator *tet*A-R was introduced into the *Salmonella* genome by selection on tetracycline resistant plates at 37°C, while the *flgJ* E223Q D248N was obtained by selection on fusaric acid plates (12 g/L Apex agar, 5 g/L Bacto tryptone, 5 g/L yeast extract, 10 g/L NaCl, 10 g/L NaH₂PO₄·H₂O, 12 µg/mL fusaric acid, 0.5 µg/mL ATc) at 42°C. Final constructs were confirmed by Sanger sequencing (Eton Biosciences).

### P22 transductions

Genetic alleles were moved between *Salmonella enterica* serovar Typhimurium strains using the generalized transducing phage P22 *HT105/1 int-201* (13). Transductions were performed by combining 100 µL of P22 transducing lysate (~10⁶ plaque-forming units/mL) prepared on donor strains with 100 µL of an overnight culture of recipient cells (2 × 10^9^ cells/mL). When necessary, the mixtures were incubated at either 37 or 30°C for 30 min to permit phenotypic expression prior to plating on selective media.

### Motility assays

Single colonies from freshly streaked strains were inoculated into soft motility agar (10 g tryptone, 5 g NaCl, 3 g Difco Bacto Agar per liter) by stabbing individual colonies with a sterile toothpick and inserting the tip into freshly poured motility plates. Plates were incubated at 37°C for 4.5 h, after which swimming halo diameters were measured and analyzed using GraphPad Prism (version 10). At least six independent colonies were tested for each genetic background.

### SDS-PAGE sample preparation and immunoblotting

Samples expressing *flhDC* under the tetracycline-inducible promoter (14) (TPOP::*flhDC*) were grown in LB medium at 37°C. At an optical density (OD₆₀₀) of 0.4, anhydrotetracycline was added, and cultures were incubated for an additional 1 h under flagellar-inducing conditions. Cell density was measured prior to harvesting. Cells were collected by centrifugation at 7,000 rpm using an Eppendorf 5430 benchtop centrifuge. Supernatants were filtered through 0.2 µm PES syringe filters to remove residual cells. Proteins from supernatant fractions were captured on 0.2 µm nitrocellulose membrane filters (13 mm diameter) and subsequently eluted by addition of SDS-PAGE loading buffer containing DTT (Bio-Rad formulation), followed by heating at 95°C for 5 min. Cell pellets were resuspended directly in SDS-PAGE loading buffer with DTT and heated at 95°C for 5 min. Samples were resolved on 16% acrylamide SDS-PAGE gels. Samples were normalized by optical density (OD), with 200 OD equivalents loaded per lane for cell pellets and 400 OD equivalents for supernatants. Proteins were transferred to Trans-Blot Turbo Mini LF PVDF membranes using the Trans-Blot Turbo Transfer System (Bio-Rad). Membranes were probed with affinity-purified polyclonal rabbit anti-FlgM or anti-FliC antibodies, followed by IRDye 800CW donkey anti-rabbit secondary antibodies (LI-COR). Signals were detected by infrared fluorescence using a Sapphire FL Biomolecular Imager (Azure Biosystems).

### Quantification of the number of flagella/cell

Flagellar number per cell was assessed by fluorescence following labeling of flagellar filaments using strains carrying a cysteine substitution at position T237 in *fliC*. Strains were locked in the FliC ON orientation (Δ*hin-5717*) to enable detection of flagella with a maleimide dye that reacts with the engineered cysteine residue in FliC. Cells were grown at 30°C to an optical density at 600 nm of ~0.6, pelleted (2 min, 800 × *g*), and gently resuspended in HEPES-buffered saline (pH 7.4; Live Cell Imaging Solution, 1×; Invitrogen) containing 0.5% paraformaldehyde. After 5 min incubation, cells were washed in HEPES-buffered saline. Flagella were labeled with DyLight 650 maleimide (Thermo Fisher Scientific) at a final concentration of 10 µM by incubating cells for 15 min in the dark. Cells were then washed in HEPES-buffered saline (2 min, 800 × *g*). Cell suspensions (4 µL) were applied to coverslips and overlaid with a 1% agarose pad as described previously (15). Imaging was performed using a Zeiss Axio Observer inverted microscope equipped with a Plan-Apochromat 100×/1.4 oil immersion objective (Ph3), YFP and CFP filter sets, an Axiocam 506 mono CCD camera, and a Colibri 7 LED light source (Zeiss). Flagella were counted manually, with ≥200 cells analyzed per strain.

### Analysis of class 2 and 3 flagellar transcriptional reporters

Strains harboring the class 2 (P*_fliF_*) and 3 (P*_fliC_*) transcriptional fusions to YFP and CFP reporters were diluted 1:100 from overnight cultures and grown in LB broth to an OD₆₀₀ of ~1. Cells were briefly pelleted, resuspended in buffered saline, and placed on ice. Cell suspensions (4 µL) were placed on large coverslips and overlaid with a 1% agarose pad as above. Imaging was performed on the Zeiss Axio Observer described above. The images analyzed contained approximately 100 individual cells/images. Mean cellular fluorescence intensity was quantified using the *Intellesis* trainable object classification module in ZEN software (version 3.12). For each genetic background, three independent biological replicates were analyzed, with 10 images acquired per replicate.

### Basal body isolation

*Salmonella* cells were grown in 500 ml of L-broth at 30°C with shaking until the cell density had reached an OD_600_ of ca. 1.0. After centrifugation (10,000 *g*, 10 min, 4°C), the cells were suspended in 20 mL of ice-cold sucrose buffer containing 0.1 M Tris-HCl (pH 8.0) and 0.5 M sucrose. EDTA and lysozyme were added at the final concentrations of 10 mM and 0.1 mg mL^−1^, respectively. The cell suspensions were stirred for 30 min at 4°C, and Triton X-100 and MgSO₄_4_ were added at final concentrations of 1% (w/v) and 10 mM, respectively. After stirring on ice for 1 h, cell lysates were adjusted to pH 10.5 with 5 M NaOH, and then centrifuged (10,000 *g*, 20 min, 4°C) to remove cell debris. After ultracentrifugation (45,000 *g*, 60 min, 4°C), pellets were resuspended in buffer A containing 10 mM Tris-HCl (pH 8.0), 5 mM EDTA, and 1% (w/v) Triton X-100, and this solution was loaded onto a 20–50% (w/w) sucrose density gradient in buffer A. After ultracentrifugation (49,100 *g*, 13 h, 4°C), basal bodies with or without hooks and filaments were collected and ultracentrifuged (60,000 *g*, 60 min, 4°C). Pellets were suspended in acidic buffer containing 50 mM glycine (pH 2.5) and 0.1% (w/v) Triton X-100 to depolymerize flagellar filaments. After ultracentrifugation (60,000 *g*, 60 min, 4°C), pellets were resuspended in 50 μL of buffer A.

### Electron microscopy

A 3 µL aliquot of isolated basal bodies was applied to a thin carbon-coated copper grid that had been glow-discharged for 20 s. Excess liquid was removed with filter paper, and the sample was stained with 2% (w/v) uranyl acetate. Grids were air-dried for at least 30 min at room temperature. Electron micrographs were acquired using a JEM-1400Flash (JEOL, Tokyo, Japan) operating at an accelerating voltage of 100 kV and recorded at a magnification of 80,000×.

### Statistical analyses

Statistical tests are carried out using GraphPad Prism. Two-tailed Student’s *t*-tests were used. Differences with *P* < 0.05 are considered significant. Data are presented as mean ± standard deviation.

## RESULTS

### Chromosomal muramidase-deficient *flgJ* mutants retain substantial motility and a large fraction of cells remain flagellated

**Fig 2:**
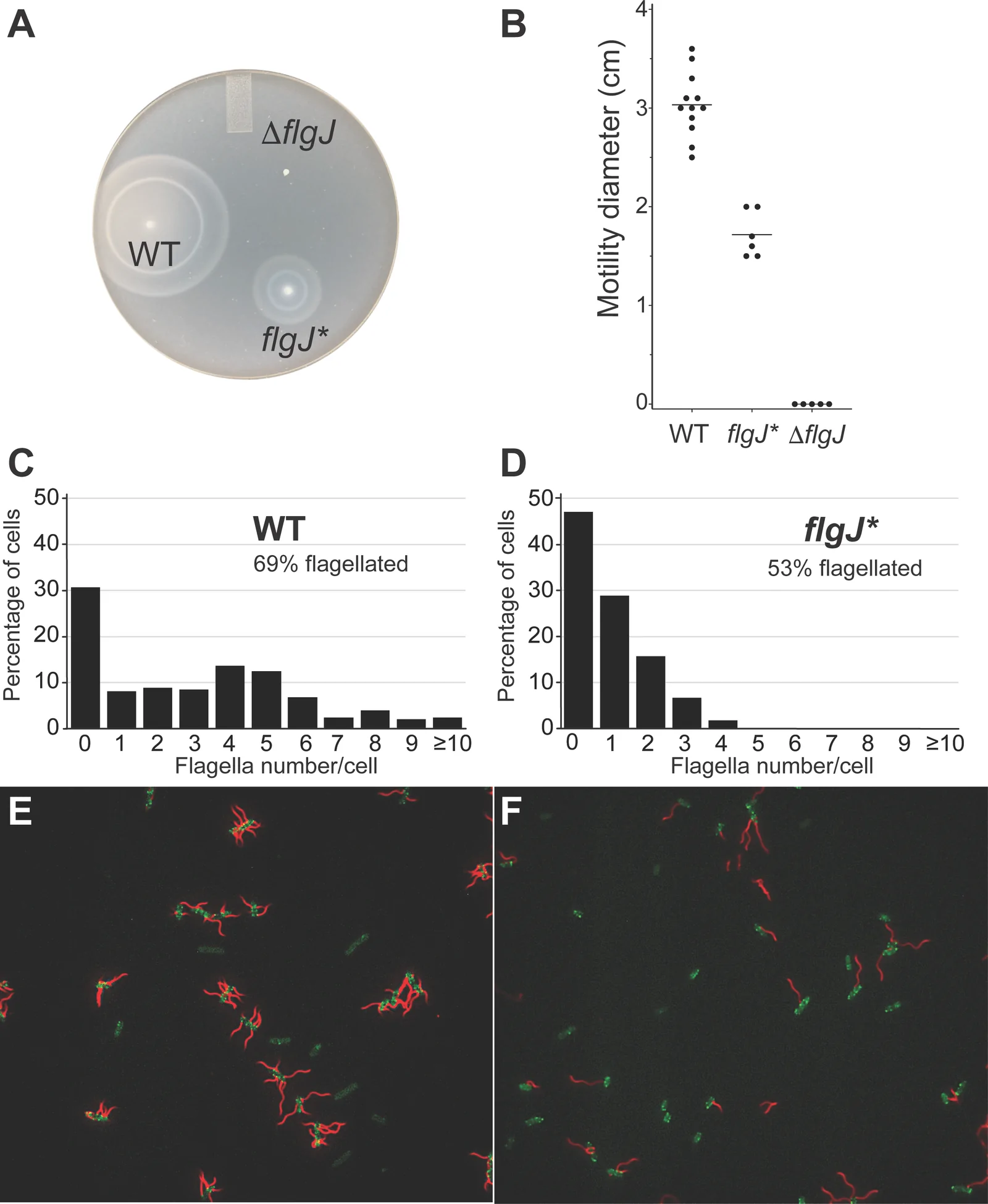
Effect of flgJ* on motility phenotypes and number of flagella/cell. (**A**) Visualization of motility phenotypes on soft agar plates. (**B**) Quantification of motility phenotypes. flgJ* showed an intermediate motility phenotype significantly different from the *flgJ*^+^ (WT) and Δ*flgJ* strains. (**C and D**) Quantification of the number of flagella/cell in the wild-type strain (**C**) and the *flgJ* mutant (**D**). The flgJ* allele results in decreased flagella number/cell compared to the wild-type strain but can still assemble complete flagella. A minimum of 200 cells were counted for each strain. Panels **E** and **F** show representative pictures from the wild-type (**E**) and flgJ* (**F**) strains. Strains used for panels **A** and **B** are LT2 (WT), TH29890 (flgJ*), and TH13962 (Δ*flgJ*). Strain used for panels **C** and **E **is TH24874 (WT), and for panels **D** and **F **is TH29920 (flgJ*).

To reassess the contribution of FlgJ catalytic activity to flagellar assembly under physiological expression conditions, we engineered a chromosomal *flgJ* allele containing two active-site substitutions, E223Q and D248N (hereafter flgJ*), to abolish acetylglucosaminidase activity (2). We found that the chromosomally encoded flgJ* strains were not as defective in motility as previously reported. They retained robust motility on soft agar with characteristic chemotaxis to serine (outer ring) and aspartate (Fig. 2A and B). The diameter of motility halos produced by the flgJ* mutant was approximately 50% of that formed by the wild-type strain, indicating that loss of FlgJ PG-hydrolyzing activity reduces, but does not eliminate, flagellar-driven motility.

To determine whether reduced motility reflected impaired flagellar assembly, we quantified the number of flagella per individual cell in these strains using fluorescence microscopy. Cells expressing the flgJ* allele produced fewer flagella per cell (Fig. 2D and F) than the wild type (Fig. 2C and E); however, approximately 50% of the flgJ* allele population remained flagellated, with many cells bearing one or more filaments (Fig. 2D and F). These observations demonstrate that penetration of the peptidoglycan layer and assembly of surface-exposed flagella occur at an appreciable frequency, even in the absence of FlgJ acetylglucosaminidase activity, albeit with reduced efficiency.

### Deletion of *flgM* increases flagella number in both the flgJ* and wild-type strains

**Fig 3:**
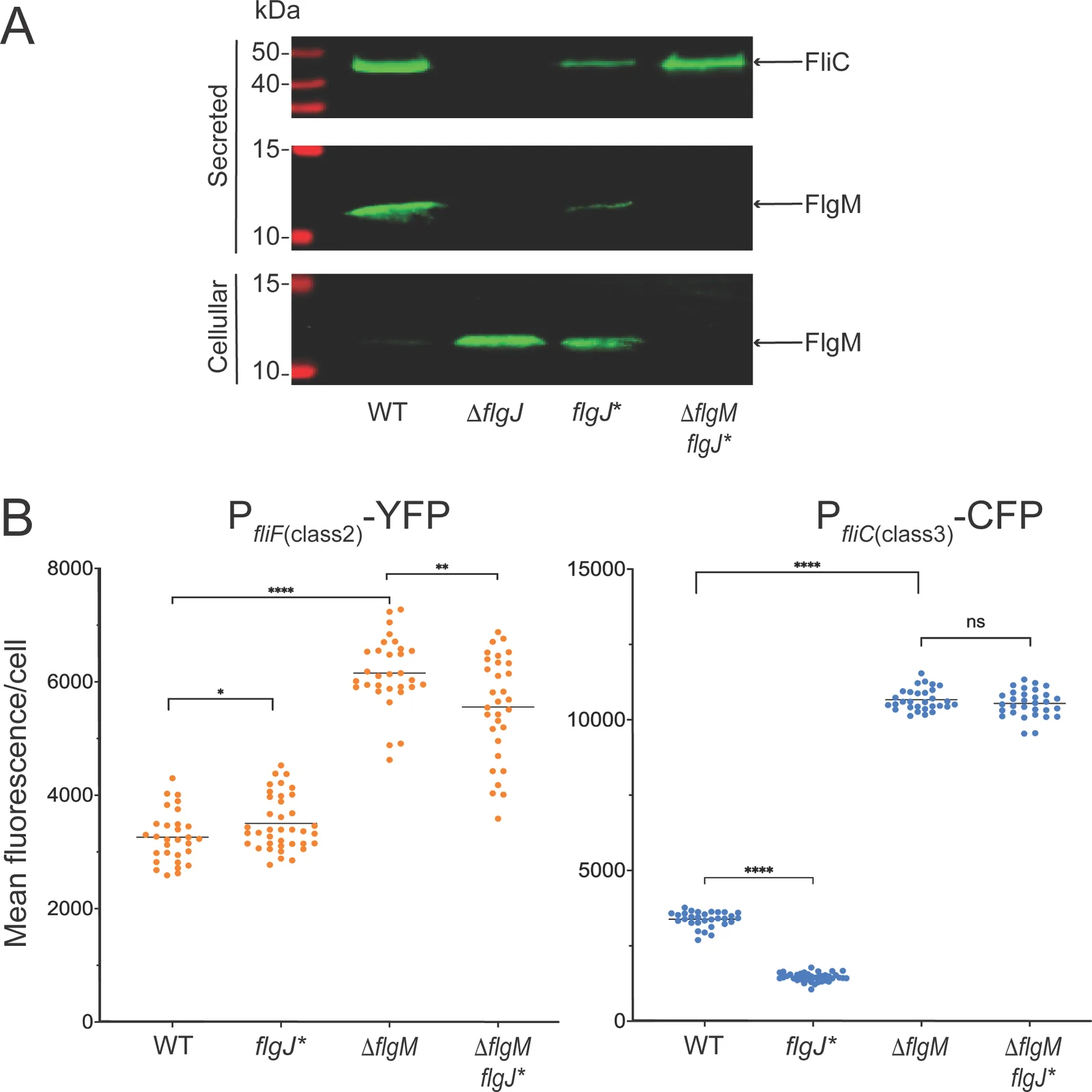
Effect of FlgM accumulation in the cellular fraction of the flgJ* mutant on class 2 and 3 flagellar gene expressions. (**A**) FlgM accumulates in the cellular fraction of the Δ*flgJ* and flgJ* mutant strains by western blot analysis. A low level of FlgM was detected in the culture supernatant of flgJ* compared to wild-type (WT) *flgJ*^+^ cells. FliC-secreted levels are also reduced in the flgJ* mutant strain. Deletion of FlgM increased FliC levels in the flgJ* mutant. Strains analyzed for panel **A** were WT (TH30535), Δ*flgJ* (TH30536), flgJ* (TH30537), and flgJ* Δ*flgM* (TH30538). (**B**) Retention of FlgM in the flgJ* strain reduces class 3 gene expression, as evident in the reduced FliC-secreted levels. Each data point represents the mean cellular fluorescence intensity (YFP or CFP) measured from a single image containing approximately 100 cells. For each genetic background, three independent biological replicates were analyzed, with 10 images acquired and quantified per replicate. The strains observed for panel **B** were the following: TH30157 (WT), TH30158 (flgJ*), TH30159 (Δ*flgM*), and TH30160 (ΔflgM flgJ*). Significant differences between groups were analyzed using two-tailed student's T-test (**** = *P* < 0.0001, ** = *P* < 0.005, * = *P* < 0.05, ns = not significant).

Mutants unable to assemble a functional HBB, including *flgJ* null mutants, are unable to secrete FlgM, which results in the inhibition of σ^28^-dependent flagellar class 3 transcription (16). As expected, we found that FlgM is secreted in a wild-type strain, resulting in class 3 transcription and FliC filament assembly, but not in a strain deleted for *flgJ* (Fig. 3A). We, therefore, asked whether the flgJ* allele led to defects in HBB formation that resulted in intracellular FlgM accumulation. This was the case. FlgM levels were greatly increased in the cellular fraction of the flgJ* mutant relative to the wild type (Fig. 3A). The intracellular accumulation of FlgM in the flgJ* allele was lower than in the Δ*flgJ* strain, consistent with a partial, rather than complete, defect in hook-basal body assembly. Deletion of *flgM* in an HBB mutant strain restores σ^28^-dependent *FliC* flagellin gene expression (16). An *flgM* deletion allele was introduced into the flgJ* background, and, consistent with the expected increase in *fliC* transcription, we observed secreted FliC levels that were similar to the secreted FliC levels observed in the wild-type strain.

Since the flgJ* allele impairs secretion of the anti-σ²⁸ factor FlgM, we analyzed the expression of class 2 and 3 promoter genes in the flgJ* allele background. Expression of fluorescence (YFP and CFP) reporters to class 2 and 3 flagellar gene promoters confirmed a selective reduction in σ²⁸-dependent (class 3) expression in the flgJ* background, while class 2 expression was not affected by flgJ* (Fig. 3B). These results suggest that impaired efficiency of basal body completion in the flgJ* background leads to partial FlgM retention and attenuated late-stage flagellar class 3 gene expression. We also noticed the effect of *flgM* deletion on class 2 activity, independent of flgJ* (Fig. 3B). Increased class 2 gene expression in the absence of FlgM has been previously reported (17), but the mechanism behind the effects of FlgM loss on class 2 flagellar gene expression has not been characterized.

**Fig 4:**
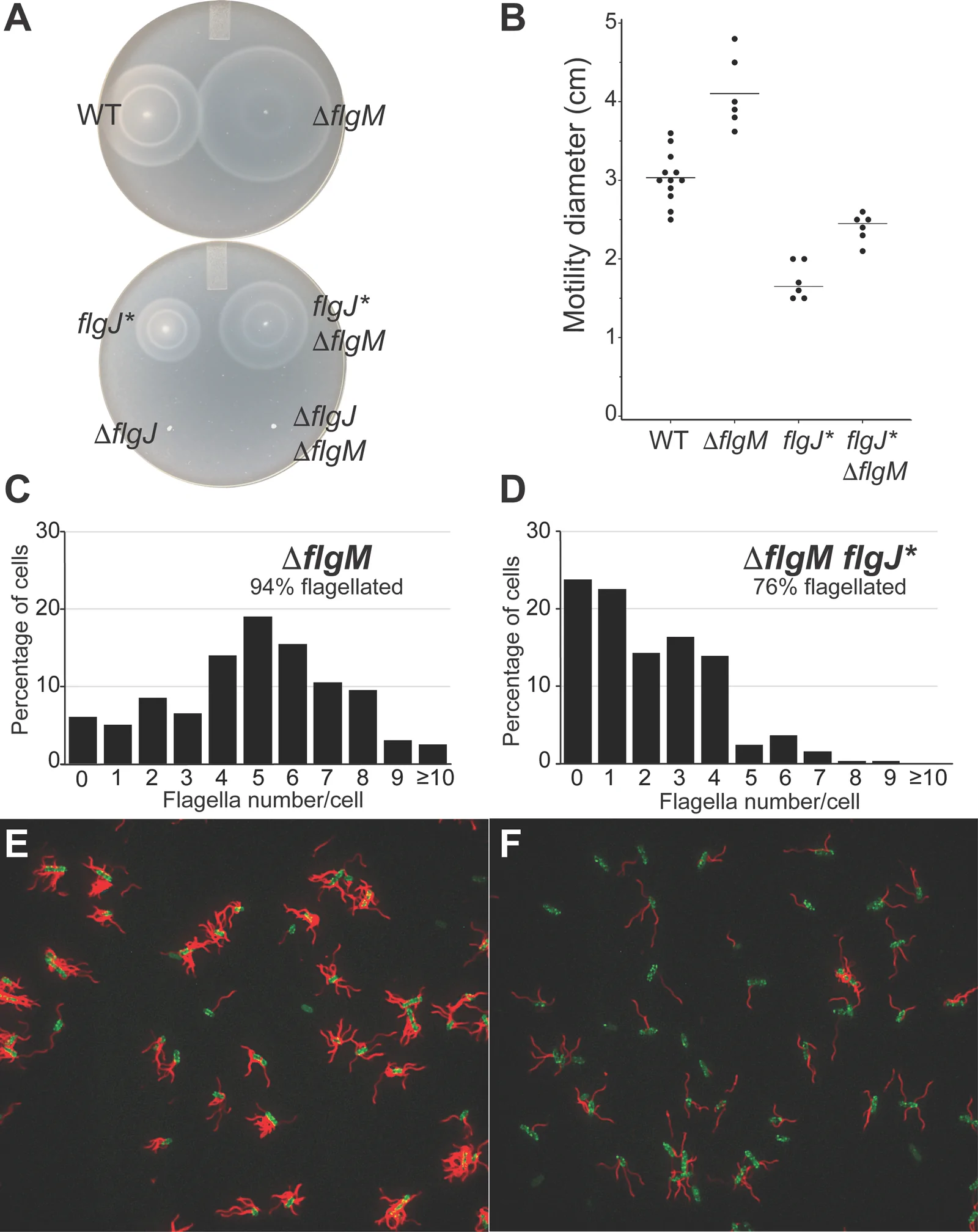
Effect of FlgM on motility and the number of flagellar/cell. (**A**) Motility on soft agar swim plates showing the effects of *flgM* and *flgJ* alleles on swimming behavior. (**B**) Deletion of *flgM* significantly increases motility in both the WT and the flgJ* mutant and (**C**) significantly increases the number of flagella per cell. Strains used for panels **A** and** B** are LT2 (WT), TH5139 (Δ*flgM*), TH29890 (flgJ*), TH30143 (flgJ*Δ*flgM*), TH13962 (Δ*flgJ*), and TH30407 (Δ*flgJ* D*flgM*). Strain used for panels** C** and **E** is TH30112 (Δ*flgM*). Strain used for panels **D** and **F** is TH30113 (flgJ* Δ*flgM*).

To determine whether FlgM accumulation contributed to the flgJ* motility phenotype, we examined the effect of deleting *flgM* in the flgJ* allele. As seen in Fig. 4A and B, removal of FlgM increased the spreading rate on soft agar plates for both *flgJ*^+^ and flgJ* strains. Deletion of *flgM* also significantly increased flagellation in both *flgJ*^+^ and flgJ* strains (Fig. 4C, D, E and F). For the flgJ* mutant, the deletion of *flgM* raised the proportion of flagellated cells from 53 to 76% (Fig. 2D and 4D). This restoration of flagellar number suggests that reduced σ²⁸ activity contributes to the motility defect in the flgJ* mutant and that increasing late gene expression can partially compensate for impaired assembly efficiency. However, deletion of *flgM* in the wild-type background similarly increased the fraction of flagellated cells (Fig. 2C and 4C) from 69 to 94% (the same increase of flagellation (around 20%) for both flgJ* and *flgJ*^+^), underscoring the strong regulatory control of FlgM on flagellar number per cell.

### Basal body structures assemble in the absence of FlgJ muramidase activity

**Fig 5:**
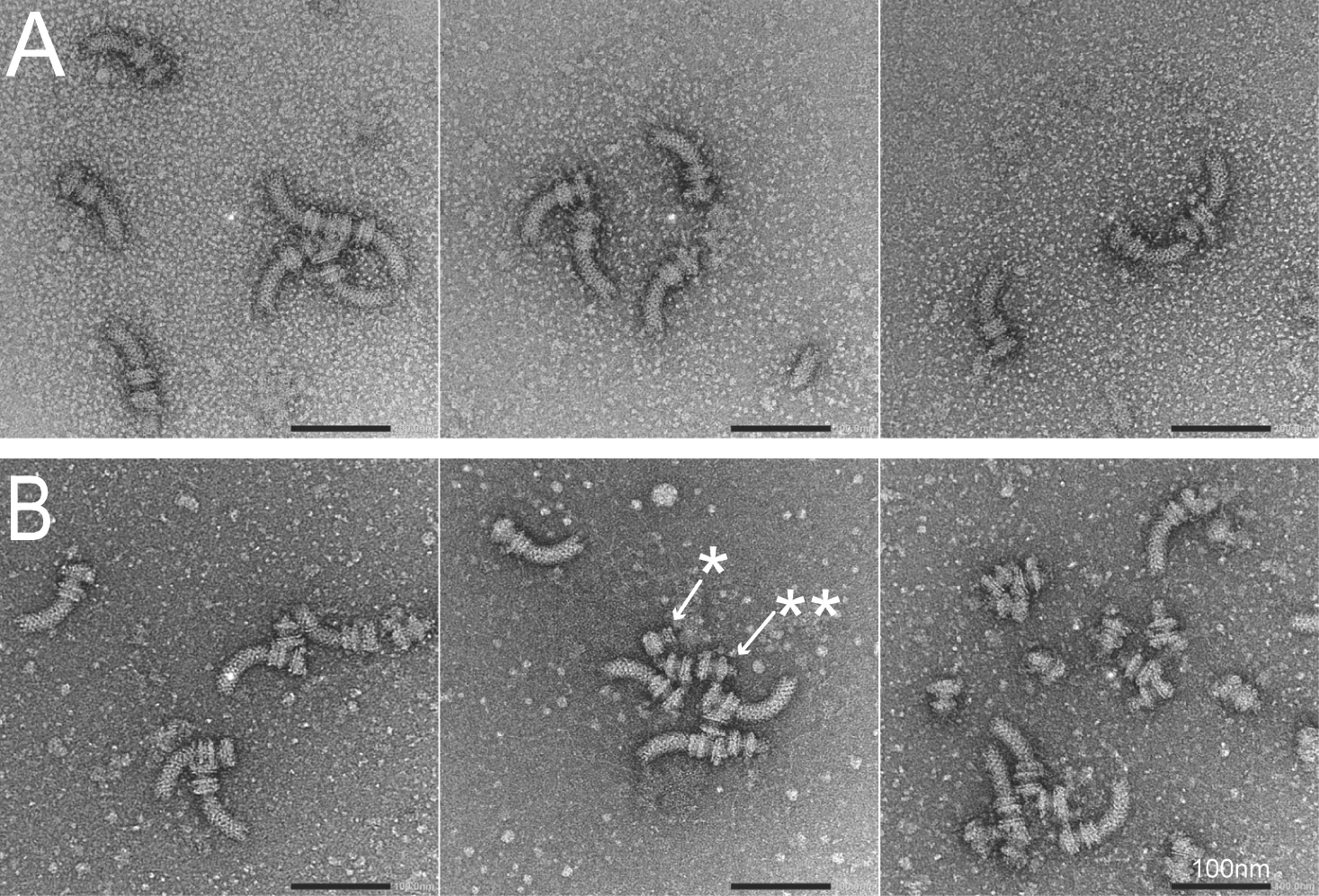
Electron micrographs of hook basal body (HBB) structures. (**A**) Cells deleted for *flgM* produce normal HBB structures. (**B**) Three different structures are observed in flgJ* Δ*flgM* double mutant strains: (i) normal HBB structures, (ii) basal structures with long rods and assembled P-rings, and (iii) basal structures with short rods lacking P-rings. Strains analyzed were TH5139 (Δ*flgM*) (panel** A**) and TH30143 (flgJ*Δ*flgM*) (panel **B**). Electron micrographs were acquired at a magnification of 80,000×. Scale bar: 100 nm. The arrows point to candlestick structures either without (*) or with (**) an attached P-ring.

To directly examine early flagellar architecture, hook-basal body complexes were isolated from wild-type and flgJ* strains and analyzed by negative-stain electron microscopy. The flgJ* mutant produced a heterogeneous population of structures, including intact HBBs with wild-type morphology and aberrant “candlestick”-like structures lacking hooks and L-rings (Fig. 5B). The presence of fully formed basal bodies and surface-exposed flagella indicates that outer membrane penetration and L-ring assembly frequently occur, even without FlgJ catalytic activity, though with reduced fidelity. The basal body structures lacking hooks appear to be of two classes, longer rod structures with added P-rings and shorter “stubs” lacking P-rings. We interpret this as varying lengths of rod polymerization into the PG layer before rod growth arrest.

## DISCUSSION

This study addresses the requirement of FlgJ’s cell wall acetylglucosaminidase activity for penetration of the peptidoglycan (PG) layer during flagellar assembly in *Salmonella*. Using chromosomally encoded catalytic mutants, we show that loss of FlgJ enzymatic activity substantially reduces, but does not abolish, flagellar assembly and motility as reported (1). The flgJ* mutant retains robust flagellar biogenesis, producing surface-exposed flagella on approximately half of the cell population. Dedicated PG hydrolysis is not universally required for flagellar assembly. Molecular data analysis identified three classes of FlgJ homologs among bacterial species: one class with a C-terminal PG-hydrolyzing domain, a second class with a C-terminal peptidase domain, and a third class with a single FlgJ scaffold domain and no added C-terminal domain (18). It was presumed that the third class utilizes an independent PG-hydrolyzing activity to allow for rod growth through the cell wall. *Caulobacter crescentus* is an example of this class with a FlgJ single domain protein and an independent PG hydrolyase, PleA, that is essential for flagellar formation (19). *Rhodobacter sphaeroides* is another example of a bacterium with a single domain FlgJ protein and independent PG-hydrolyzing protein, SltF, that is essential for rod polymerization through the cell wall (20, 21). *Bacillus subtilis* appears to be an exception. *B. subtilis* lacks either a C-terminal PG hydrolase or peptidase domain despite possessing a substantially thicker cell wall than gram-negative organisms. Although several σ^D^-controlled autolysins (LytC, LytD, and LytF) have been implicated in PG remodeling, deletion of these enzymes does not abolish flagellar assembly (22), and additional PG hydrolases, such as CwlQ, contribute primarily to motility behaviors rather than basal body formation (23). Moreover, genome-wide transposon mutagenesis studies in *B. subtilis* have failed to identify any FlgJ-like factor essential for flagellar-dependent motility (24). Recent interrogation of the *B. subtilis* cell wall by atomic force microscopy indicates that it is more porous than previous estimates (25, 26). Furthermore, expression of a highly expressed large (99.3 kDa) fusion protein with a Sec secretion signal resulted in its efficient secretion into the medium, leading the authors to propose the existence of protein secretion zones (27). Together, these observations suggest that bacteria employ diverse and partially redundant strategies for PG remodeling, and that the physical properties of the PG layer itself may permit rod penetration without a dedicated hydrolase. In the absence of catalytic activity, assembly may proceed more slowly or stochastically, relying on basal PG turnover, redundant envelope hydrolases, or transient openings within the PG meshwork, as recently seen with *Bacillus* (11).

**Fig 6:**
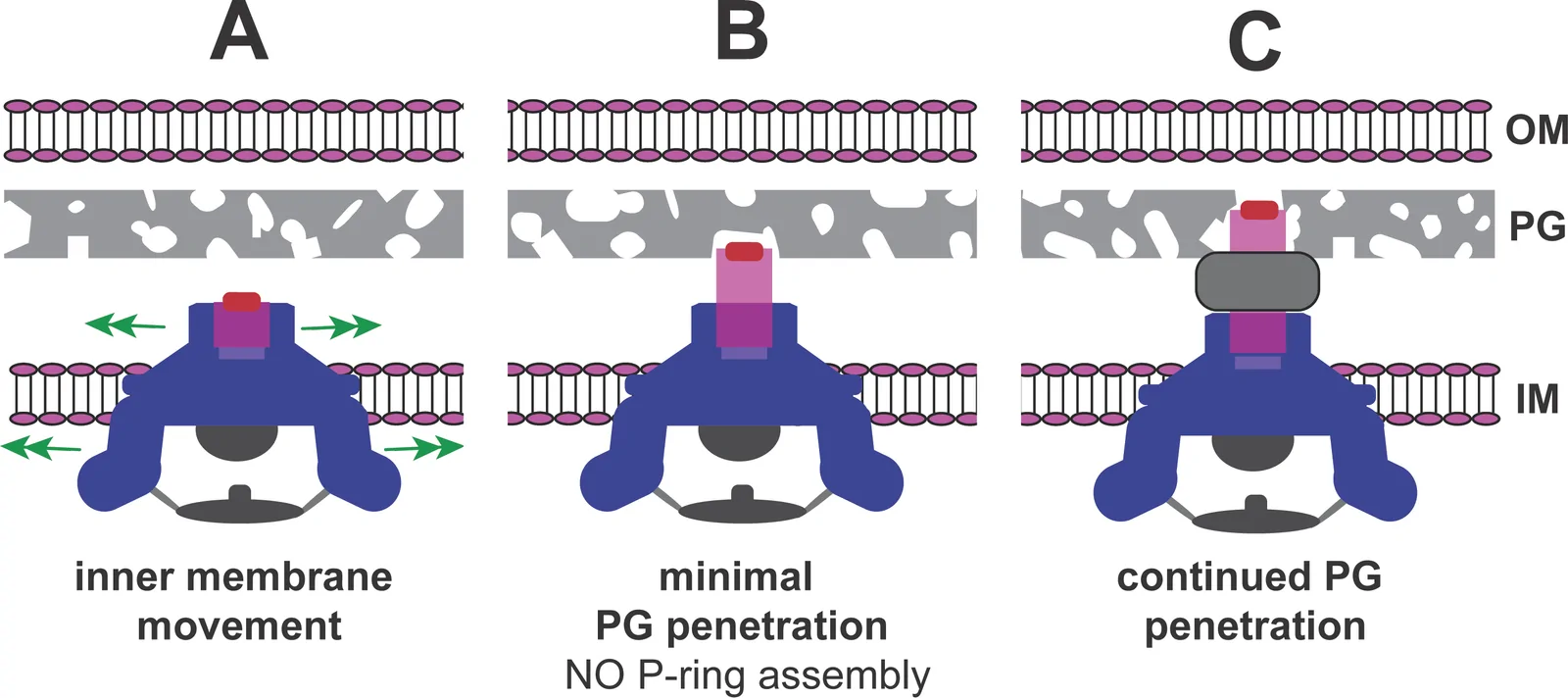
A model explaining why flgJ* mutants can produce “candlesticks” or continue rod assembly. The model is based on the prediction from an earlier study for the existence of permissive holes in the peptidoglycan (PG) that allow for partial and complete rod assembly independent of PG hydrolysis (1). (**A**) Prior to peptidoglycan (PG) penetration, basal bodies are mobile within the inner membrane. (**B**) Small openings in the PG layer permit partial rod assembly; however, assembly stalls when the PG is too thick to be penetrated. (**C**) In some cases, the growing rod encounters a locally thinner region of PG, allowing assembly to proceed.

The strong restoration of flagellation observed upon deletion of *flgM* demonstrates that the flagellar assembly pathway remains fundamentally competent in the absence of FlgJ PG-hydrolyzing activity. Increased σ²⁸-dependent expression of late flagellar genes compensated for reduced assembly efficiency by enhancing filament production and increasing the probability that completed hook-basal bodies acquire filaments. These results support a kinetic model in which FlgJ-mediated PG hydrolysis accelerates or biases rod penetration toward productive outcomes but only when the PG is encountered. The *Salmonella* PG is porous to the extent that a significant number of rods can penetrate the PG without the hydrolyzing activity of FlgJ (Fig. 6).

Our findings also provide insight into the regulatory logic underlying flagellar biogenesis. Deletion of *flgM* resulted in hyper-flagellation and imposed a measurable growth cost, consistent with previous work showing that excessive basal body formation and filament synthesis can be deleterious (4, 28, 29). This suggests that FlgM-mediated inhibition of σ²⁸ serves not merely as a checkpoint for assembly completion, but as a mechanism to limit the number of flagella produced per cell. Such regulation would be under strong evolutionary pressure to ensure the most efficient number of peritrichous flagella are synthesized and assembled and might also prevent the accumulation of nonproductive or incomplete structures. In this context, FlgM may act to optimize the balance between motility and fitness by coupling late-stage gene expression to successful basal body assembly.

In summary, our results reveal that FlgJ-mediated PG hydrolysis enhances the efficiency of the assembly of the basal body but is not strictly required for rod penetration or flagellar biogenesis in *Salmonella*. During flagellum assembly, *Bacillus* lacks a PG-hydrolyzing rod cap; thus, its thick gram-positive PG layer must permit penetration through intrinsic porosity rather than enzymatic remodeling. These findings refine the classical model of flagellar assembly and highlight the robustness, flexibility, and evolutionary diversity of the mechanism bacteria use to overcome potential PG barrier to rod penetration during construction of complex envelope-spanning structures.
